# MEDiCINe: Motion Correction for Neural Electrophysiology Recordings

**DOI:** 10.1523/ENEURO.0529-24.2025

**Published:** 2025-03-04

**Authors:** Nicholas Watters, Alessio Buccino, Mehrdad Jazayeri

**Affiliations:** ^1^Department of Brain and Cognitive Sciences, Massachusetts Institute of Technology, Cambridge, Massachusetts 02139; ^2^ McGovern Institute of Brain Research, Massachusetts Institute of Technology, Cambridge, Massachusetts 02139; ^3^Allen Institute for Neural Dynamics, Seattle, Washington 98109; ^4^CatalystNeuro, Benicia, California 94510; ^5^ Howard Hughes Medical Institute, Chevy Chase, Maryland 20815

**Keywords:** electrophysiology, machine learning, motion correction, Neuropixels, primate, spike sorting

## Abstract

Electrophysiology recordings from the brain using laminar multielectrode arrays allow researchers to measure the activity of many neurons simultaneously. However, laminar microelectrode arrays move relative to their surrounding neural tissue for a variety of reasons, such as pulsation, changes in intracranial pressure, and decompression of neural tissue after insertion. Inferring and correcting for this motion stabilizes the recording and is critical to identify and track single neurons across time. Such motion correction is a preprocessing step of standard spike-sorting methods. However, estimating motion robustly and accurately in electrophysiology recordings is challenging due to the stochasticity of the neural data. To tackle this problem, we introduce **MEDiCINe** (**M**otion **E**stimation by **Di**stributional **C**ontrastive **I**nference for **Ne**urophysiology), a novel motion estimation method. We show that MEDiCINe outperforms existing motion estimation methods on an extensive suite of simulated neurophysiology recordings and leads to more accurate spike sorting. We also show that MEDiCINe accurately estimates the motion in primate and rodent electrophysiology recordings with a variety of motion and stability statistics. We open-source MEDiCINe, usage instructions, examples integrating MEDiCINe with common tools for spike sorting, and data and code for reproducing our results. This open software will enable other researchers to use MEDiCINe to improve spike sorting results and get the most out of their electrophysiology datasets.

## Significance Statement

Recent advances in high-density microelectrode arrays such as Neuropixels have allowed neurophysiologists to record from hundreds of neurons simultaneously. Such data scale necessitates automatic isolation and tracking of individual neurons throughout a recording session, a process called “spike sorting.” One challenge for automated spike-sorting algorithms is relative motion between the electrodes and the brain, which must be corrected to stabilize the recording. We introduce a method for estimating such motion in neural recordings. Our method outperforms existing motion estimation methods and produces more accurate spike sorting on a benchmark of simulated datasets with known ground-truth motion. Our method also performs well on primate neurophysiology datasets. We open-source our method and instructions for integrating it into common spike-sorting pipelines.

## Introduction

**Figure 1.**
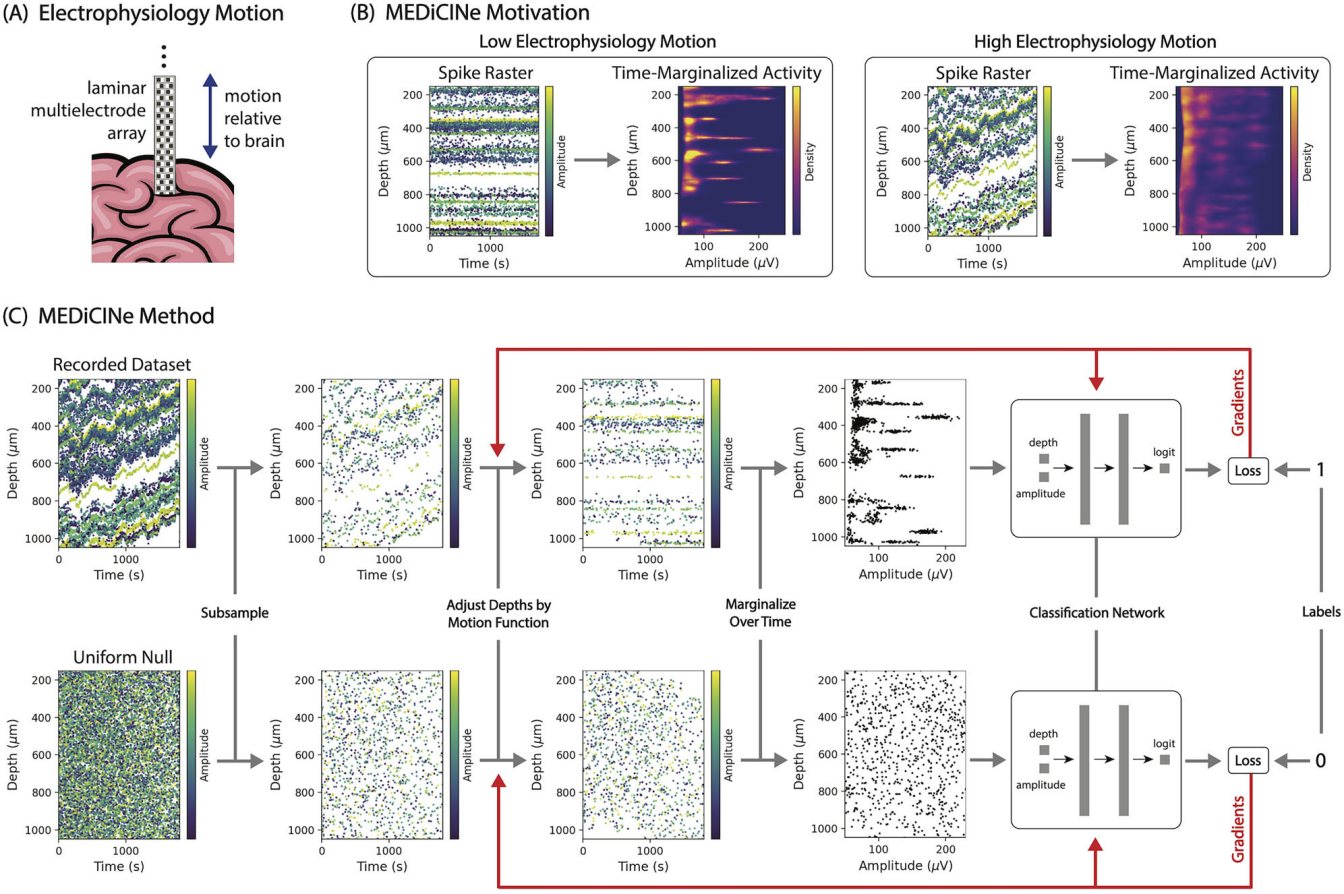
MEDiCINe is a method for estimating motion in electrophysiology data. ***A***, Laminar multielectrode array (checkerboard) inserted into the brain (pink) moves relative to the brain over time in a recording session. ***B***, In recordings with low electrophysiology motion, neural activity does not vary in depth over time, causing time-marginalized activity to be sparse in depth-amplitude space. In recordings with high electrophysiology motion, neural activity varies in depth over time, causing time-marginalized activity to not be sparse in depth-amplitude space. ***C***, MEDiCINe motion estimation procedure. For each training step, spikes are sampled from the recorded dataset and from a uniform null distribution. Each spike has a time, depth, and amplitude feature. Spike depths are adjusted by a differentiable motion function and then marginalized over time. A classification network then discriminates between spikes that came from the dataset and spikes that came from the uniform distribution. Binary cross-entropy classification loss is backpropagated to update the parameters of both the classification network and the motion function.

Electrophysiology studies often involve recording neural activity with laminar microelectrode arrays inserted in the brain. This data is processed to compute putative spike times of individual neurons throughout a recording session, a process termed “spike sorting.” Historically, spike sorting was primarily a manual or semimanual process (Wilson and McNaughton, 1993; Schmitzer-Torbert et al., 2005; Plexon, Inc, 2017). However, recent advances in recording scale afforded by high-density laminar microelectrode arrays such as Neuropixels probes (Jun et al., 2017) have made manual spike sorting prohibitively time-consuming. This has necessitated the emergence of automated spike-sorting algorithms (Buccino et al., 2022). Automating spike sorting is challenging for several reasons, one of which is that the laminar microelectrode array (hereafter referred to as “array”) may move relative to its surrounding neural tissue (Fig. 1*A*; Steinmetz et al., 2018; Garcia et al., 2024). This motion can be caused by a variety of factors, such as pulsation, changes in intracranial pressure, decompression of neural tissue after inserting an array, and instability of the mechanical apparatus holding the array. Motion is typically more extreme in nonhuman primate (NHP) and human recordings than recordings in rodents and other small animals (Coughlin et al., 2023; Windolf et al., 2023). Estimating and correcting for motion is an important step in spike-sorting pipelines. Improvements in motion estimation yield better automatic spike sorting, both yielding more usable neurons for analysis and saving the researcher time manually curating spike-sorting results (Garcia et al., 2024).

Electrophysiology motion estimation is challenging for several reasons. First, the motion can exhibit a range of statistics, including slow drift, high-frequency noise, and discrete jumps. Second, the motion may depend on position along the array, varying as a function of depth in the brain. Third, the neural activity itself may be nonstationary: Neuron firing rates may fluctuate over time, neurons may be gained or lost throughout the session due to motion or cell death, and the relative motion between the array and the brain may cause the recorded waveform shape of single neurons to change.

Existing state-of-the-art approaches to motion estimation begin by discretizing the data into temporal bins (Windolf et al., 2023; Pachitariu et al., 2024). For each temporal bin, they compute a histogram of neural activity as a function of depth along the array and neural activity features such as waveform amplitude. An estimate of the motion is computed to maximize the correlations across pairs of these histograms. This computation may involve comparing each temporal bin to a particular reference bin (Pachitariu et al., 2024) or may involve comparing each temporal bin only to its nearest neighbors (Windolf et al., 2023). While these approaches work well for some recordings, there is broad agreement in the field that existing approaches struggle for some recordings and accurate motion estimation is a common difficulty for spike sorting.

We introduce **MEDiCINe** (**M**otion **E**stimation by **Di**stributional **C**ontrastive **I**nference for **Ne**urophysiology), an approach for motion estimation that infers motion by fitting a constrained model of the neural data. We first consider the generative process of the neural data, which has two components: (1) neural activity consisting of local voltage modulations, such as spikes or LFP power, coming from neurons that are unmoving in the brain tissue, and (2) motion of the array relative to the brain. We then formulate a nonparametric model of neural activity that captures this generative structure. We fit this model to neural data using gradient descent. We found that this approach works on a wide range of datasets without hyperparameter tuning. MEDiCINe outperforms existing methods on an extensive suite of simulated datasets with known ground-truth motion and a variety of motion and instability statistics. MEDiCINe also works well on all of our NHP Neuropixels recordings and on rodent Neuropixels recordings with experimentally imposed motion. Lastly, we open-source MEDiCINe, usage instructions, examples integrating MEDiCINe with SpikeInterface and Kilosort4 tools for spike sorting, and data and code for reproducing our results.

## Materials and Methods

### MEDiCINe method

Consider a dataset of N spikes (putative action potentials) extracted from an electrophysiology recording session. Represent the dataset as a set of triples $\left[\left(t_{1},d_{1},a_{1}),\;(t_{2},d_{2},a_{2}),\;\ldots ,\;(t_{N},\;d_{N},\;a_{N}\right)\right]$, where $t_{i}$ is the time at which spike i occurred, $d_{i}$ is the estimated depth along the laminar array at which spike i was detected, and $a_{i}$ is the amplitude of spike i. If the recording has low electrophysiology motion through time, then marginalizing this dataset over time would yield a sparse distribution in depth–amplitude space, under the assumption that individual neurons have stable depth and spike amplitude in the brain. In contrast, if the recording has high motion, then marginalizing this dataset over time would not yield a sparse dataset, because spikes coming from a single neuron would be spread out over depth (Fig. 1*B*). Leveraging this observation, the key intuition underlying MEDiCINe is to learn a motion function that maximizes the sparsity of the time-marginalized dataset distribution (Fig. 1*C*). The following architecture and objective function of MEDiCINe formulate this sparsity maximization in a way that facilitates computationally efficient optimization.

To operationalize this, MEDiCINe learns two differentiable functions:

MotionFunctionM:(d,t)→Δd,

ClassificationNetworkC:(d,a)→p∈[0,1].

These functions compose to form a probability function P over the joint space [time, depth, amplitude]:

P(t,d,a)=C(d+M(d,t),a).

P is trained to classify whether an input spike comes from the dataset or from a uniform null distribution over depth and amplitude. This pressures the motion-corrected dataset spikes d+M(d,t) to be a sparse distribution that is highly discriminable from a uniform null. Specifically, M and C are fit using gradient descent. For each step, we draw a batch $\left[\left(t_{i_{1}},\;d_{i_{1}},a_{i_{1}}),\;\ldots ,\;(t_{i_{K}},\;d_{i_{K}},\;a_{i_{K}}\right)\right]$ of K random samples from the spike dataset and a batch $\left[\left({\hat{t}}_{\;j_{1}},{\hat{d}}_{\;j_{1}},{\hat{a}}_{\;j_{1}}),\;\ldots ,\;({\hat{t}}_{\;j_{K}},{\hat{d}}_{\;j_{K}},{\hat{a}}_{\;j_{K}}\right)\right]$ of K random samples from a uniform distribution with the same ranges at the spike dataset. We then apply P to each of these batches to get $[\;p_{i_{1}},\ldots ,\;p_{i_{K}}]\;$and $\left[{\hat{p}}_{\;j_{1}},\;\ldots ,{\hat{p}}_{\;j_{K}}\right]$, where $p_{i_{l}}=P(t_{i_{l}},\;d_{i_{l}},\;a_{i_{l}})$ and ${\hat{p}}_{\;j_{l}}\;=\;P({\hat{p}}_{\;j_{l}},\;{\hat{d}}_{\;j_{l}},\;{\hat{a}}_{\;j_{l}})$ . We then compute the loss function as follows:

$$L\;=\;\overset{K}{\underset{l=1}{\sum }}\;\log\;(\;p_{i_{l}})+\log(1-{\hat{p}}_{\;j_{l}}).$$

This is the binary cross-entropy loss where the data samples are labeled 1 and the uniform samples are labeled 0. We backpropagate L to update the parameters in M and C. This loss function pressures P to discriminate dataset samples from uniform samples, hence pressuring the motion function to cause the time-marginalized dataset distribution after motion adjustment to be sparse. We parameterize the classification network C by a multilayer perceptron with two hidden layers. We parameterize the motion function M as the linear interpolation of a matrix of shape [depth bins, time bins] discretizing the space of depth and time. The entries of this matrix are Δd estimates. Using multiple depth bins allows the motion function to model motion that varies across depth.

Note that this method is not specific to datasets with only [time, depth, amplitude] representations of spikes. It can apply more generally to any dataset of neural events that have a [time, depth, feature vector] representation. This includes spike data with spike shape features beyond amplitude and LFP data with power spectrum features. Note also that this method is not specific to motion only in depth. By letting the motion function return a three-vector [Δ*x*, Δ*y*, Δ*z*], it could estimate motion in three-dimensional space.

### Code and data accessibility

To use MEDiCINe, please visit our MEDiCINe website https://jazlab.github.io/medicine. That website includes demos and instructions and for using MEDiCINe on your own data, including interfacing with Kilosort4 and SpikeInterface.

To reproduce the results in this work, please visit https://github.com/jazlab/medicine_paper for software, data, and instructions for reproducing the results in this manuscript.

## Results

To evaluate MEDiCINe and compare it to existing motion estimation methods, we quantitatively benchmarked it on a suite of simulated datasets with controlled ground-truth motion. We also qualitatively assessed its performance on NHP electrophysiology datasets without known ground-truth motion.

### Simulated datasets

To compare the performance of MEDiCINe with existing motion estimation methods, we generated a suite of 384 simulated neurophysiology recording datasets with controlled ground-truth motion and evaluated both MEDiCINe and existing motion estimation methods on these datasets. To generate the simulated datasets, we enlarged a preexisting suite of simulated datasets (Garcia et al., 2024) to include a wide variety of motion and neuron stability statistics that occur in neurophysiology data. We used the MEArec electrophysiology data simulator (Buccino and Einevoll, 2021) to generate one 30 min Neuropixels electrophysiology session for each combination of the following dataset parameters:

- Linear drift of the relative depth between the array and brain. Two options: (1) no linear drift or (2) linear drift of 0.1 μm/s.
- Random walk of the relative depth between the array and brain with Gaussian steps and 1 s frequency. Three options: (1) no random walk, (2) random walk with standard deviation of 1 μm/s, or (3) random walk with standard deviation of 2 μm/s.
- Discrete random jumps of the relative depth between the array and brain. Two options: (1) no jumps or (2) jump times sampled from a Poisson process with a rate of 100 *s* and jump displacements sampled from a uniform distribution over [−50 μ*m*, 50 μ*m*].
- Number of neurons. Two options: (1) 20 neurons or (2) 100 neurons.
- Distribution of neuron density over depth. Two options: (1) neurons uniformly distributed over depth or (2) neurons distributed bimodally over depth from a mixture of two Gaussians with means at 15 and 85% of the array length and standard deviations of 10% of the array length.
- Firing rate stability. Four options: (1) constant firing rates randomly uniformly sampled between 1 and 20 Hz, (2) periodic firing rates that are synchronous over all neurons with a period of 4 min and a mean of 1.5 Hz, (3) periodic firing rates that are asynchronous over all neurons, or (4) half of the neurons have constant firing rates, while the other half appear or disappear at random times in the session with linearly ramping firing rate between 0 Hz and a random maximum value between of 1 and 20 Hz.
- Depth dependency of motion. Two options: (1) depth-independent (rigid) motion or (2) depth-dependent (nonrigid) motion that varies linearly over depth with coefficient 1 for the deepest electrode and 0.5 for the shallowest electrode.

**Figure 2.**
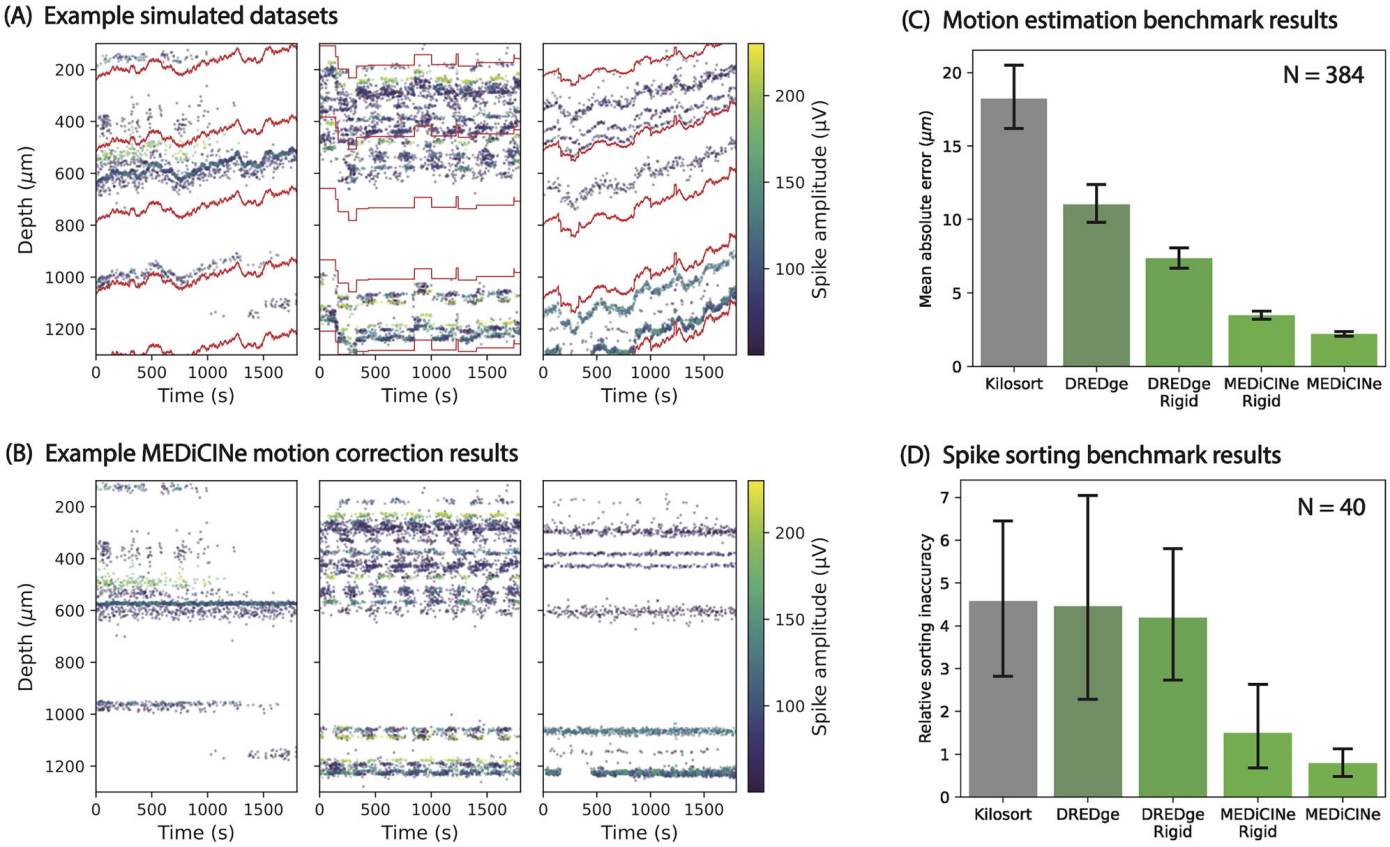
Simulated data results. ***A***, Examples for 3 of our 384 simulated datasets used for benchmarking. Each point represents a detected spike. Only 3% of spikes are shown. Red lines show the ground-truth motion, namely, the depth on the array over time of stationary points in the brain. The left dataset has random walk motion with a standard deviation of 2 μm/s, 20 neurons, and firing rate instability consisting of appearing/disappearing neurons. The middle dataset has motion consisting of discrete random jumps, 100 neurons with bimodal density over depth, and asynchronous periodic firing rate instability. The right dataset has depth-dependent motion consisting of both linear drift and random walk with a standard deviation of 2 μm/s and 20 neurons with bimodal density over depth. ***B***, Spike raster plots after correcting for motion estimated by MEDiCINe for the three example datasets in (***A***). ***C***, Mean absolute error (*y*-axis) between estimated motion and ground-truth motion over all 384 simulated datasets for each motion estimation method (*x*-axis). Error bars show 95% confidence intervals of the mean. Kilosort, 18.23 ± 2.12; DREDge, 11.03 ± 1.32; DREDge Rigid, 7.36 ± 0.69; MEDiCINe Rigid, 3.48 ± 0.28; and MEDiCINe, 2.21 ± 0.17. Extended Data Figure 2-1 shows a breakdown of these results conditionalized on each factor of variation of the datasets. Extended Data Figure 2-4 shows worst-case results of all methods. ***D***, Mean relative spike-sorting inaccuracy (*y*-axis) over 40 simulated datasets between ground-truth spike times per unit and Kilosort4 estimated spike times per unit after motion estimation for each motion estimation method (*x*-axis). Error bars are 95% confidence intervals of the mean. Kilosort, 4.57 ± 1.87; DREDge, 4.46 ± 2.33; DREDge Rigid, 4.19 ± 1.61; MEDiCINe Rigid, 1.50 ± 0.94; and MEDiCINe, 0.79 ± 0.34. Extended Data Figure 2-2 shows a distributional view of the results in (***C***) and (***D***), including outliers. Extended Data Figure 2-3 shows an analysis of how the methods rank relative to each other across datasets.

From these datasets we extracted spike times, estimated depths, and amplitudes using the monopolar triangulation method (Buccino et al., 2018; Boussard et al., 2021). Figure 2*A* shows spike raster plots of three example simulated datasets and Figure 2*B* for results of MEDiCINe applied to these datasets. We evaluated the following five motion estimation methods on the extracted spikes from each of our 384 simulated datasets:

- Kilosort. The “datashift” motion estimation function from Kilosort4 (Pachitariu et al., 2024) with default parameters, which is currently the most recent motion estimation in the Kilosort family.
- DREDge. The official DREDge implementation in the SpikeInterface library version 0.101.2 (Buccino et al., 2020) with default parameters, currently considered the state-of-the-art motion estimation method (Windolf et al., 2023).
- DREDge Rigid. A modification of DREDge that enforces rigid motion as a function of depth and uses center-of-mass depth estimation instead of monopolar triangulation (Garcia et al., 2024). This is implemented as the “rigid_fast” method in SpikeInterface version 0.101.2.
- MEDiCINe Rigid. Our MEDiCINe method with a single depth bin, enforcing rigid motion as a function of depth.
- MEDiCINe. Our MEDiCINe method with multiple depth bins. In practice, we used two depth bins, which is the same number as DREDge uses on our simulated datasets with the default parameters.

10.1523/ENEURO.0529-24.2025.f2-1 Figure 2-1 Results on Simulated Data Conditioned on Parameters. Motion estimation model results conditioned on each parameter of variation of simulated dataset suite. Errorbars show 95% confidence interval of the mean. Left column shows mean absolute error, and right column shows method ranking. Download Figure 2-1, TIF file.

10.1523/ENEURO.0529-24.2025.f2-2 Figure 2-2 **Benchmark Violin Plots (A)** A violin plot representation of the results in Figure 2-A. **(B)** A violin plot representation of the results in Figure 2-B. Download Figure 2-2, TIF file.

**Figure 3.**
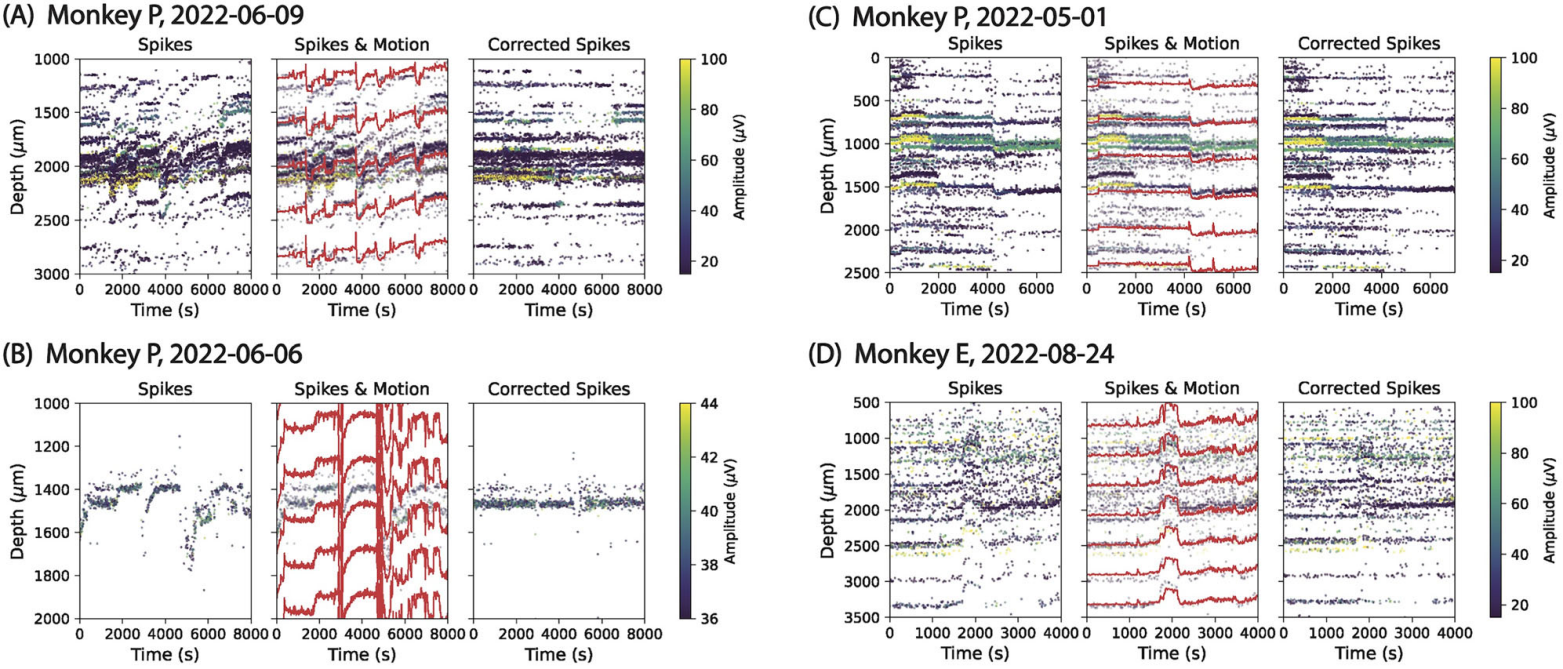
Primate data results. ***A*–*D***, Each panel represents a different NHP Neuropixels dataset that we recorded, showing a raster plot of spikes (left), interpolated motion curves estimated by MEDiCINe (red lines) overlayed on the raster plot (middle), and the raster plot after correcting by the motion estimated by MEDiCINe (right). Extended Data Figure 3-1 for results of the other motion estimation methods on these datasets. Extended Data Figure 3-2 for results on rodent neurophysiology datasets.

10.1523/ENEURO.0529-24.2025.f2-3 Figure 2-3 **Benchmark method Rankings (A)** For each simulated dataset, we compute the ranking (1-5) of each of the 5 motion estimation methods on that dataset in terms of mean absolute motion estimation error. This ranking is shown on the y-axis. **(B)** For each simulated dataset for which we run spike sorting, we compute the ranking (1 - 5) of each of the 5 motion estimation methods on that dataset in terms of relative sorting inaccuracy. This ranking is shown on the y-axis. Download Figure 2-3, TIF file.

10.1523/ENEURO.0529-24.2025.f2-4 Figure 2-4 **Failure Cases (A)** Kilosort motion estimation results for the simulated dataset for which the difference between Kilosort and the best method is greatest. This represents the worst failure case for Kilosort in our suite of simulated datasets. **(B) - (E)** Corresponding failure cases for the other methods. Download Figure 2-4, TIF file.

10.1523/ENEURO.0529-24.2025.f2-5 Figure 2-5 **Spike Sorting Accuracy.** Accuracy as a function of unit (sorted by accuracy) for Kilosort4 sorting results for each motion estimation method on each of the 40 datasets for which we ran spike sorting. Download Figure 2-5, TIF file.

### MEDiCINe implementation

We parameterized the motion function of MEDiCINe by an array of size [*depth_bins, time_bins*]. For multiple depth bins, the depth bins uniformly divided the range from the deepest to the shallowest detected spike. We let *time_bins* equal the ceiling of the number of seconds in the dataset, allowing the model to capture motion at 1 s resolution. We also applied a triangular temporal smoothing kernel with 30 s support. We found this temporal resolution and smoothness to be sufficiently fine to capture motion well in all our datasets. To compute the change in depth at a given time and depth, we computed the linear interpolation of the temporally smoothed motion array for that time and depth. We then applied a scaled hyperbolic tangent function to bound the motion by ±400 μ*m*.

We parameterized the activity network of MEDiCINe by a multilinear perceptron with 14 input units, two fully connected hidden layers each with 256 units, and one output unit. The activation function was ReLU. We applied a sigmoid function to the output to force it to be a probability in [0, 1]. Given a depth and amplitude, to compute the probability of a corresponding spike, we did the following:

- Normalize both the depth and amplitude to lie in [0, 1], given the depths and amplitudes of all spikes in the dataset.
- Compute six depth features by taking sin(x⋅depth) for *x* in [1, 2, 4, 8, 16, 32]. Similarly, compute six amplitude features.
- Concatenate the depth and amplitude with their features into a 14-dimensional vector.
- Apply the MLP to this vector.

We added the sinusoidal features as inputs to the network because they helped optimization by allowing the MLP to more easily learn high-frequency modulations. In our experiments, these features improved optimization convergence runtime by about a factor of 10.

We implemented the model in PyTorch (Paszke et al., 2019) and trained it with the Adam optimizer (Kingma, 2014) with a learning rate of 5 · 10^−4^ and gradient clipping of 1. We used batch size 8,192, where each batch had 4,096 spikes randomly sampled from the dataset and 4,096 spikes randomly sampled from a uniform distribution with the same depth, amplitude, and time bounds as the spike dataset. We trained for 10,000 gradient steps. To reduce the chance of converging to a local minimum, we added noise to the motion function output early in training. At the start of training, this noise had standard deviation equal to 0.1 times the depth range of the data. This was linearly annealed to 0 throughout the first 2,000 gradient steps of training.

### Benchmark results

We evaluated performance of all motion estimators using a standard measure of the median-corrected mean absolute error with respect to the ground-truth motion (Garcia et al., 2024). Specifically, for each dataset, we selected 11 depth levels evenly spaced from the deepest to the shallowest recorded spike. For each of these depth levels and each model, we computed the ground-truth motion *M* through time at 1 s intervals and the motion $\tilde{M}$ estimated by the model at 1 s intervals. For each level, we compute the median-corrected absolute difference $\mathrm{abs}(M-\;\tilde{M}-\;\mathrm{median}(M-\;\tilde{M}))$. The model's mean absolute error is the average of this quantity over time and depth levels (Garcia et al., 2024). By this metric, MEDiCINe Rigid and MEDiCINe significantly outperformed all other methods on average (Fig. 2*C*). When conditioning these results on each factor of variation of the datasets, MEDiCINe always performed at least as well as all existing methods (Extended Data Fig. 2-1). These results are not due to outlier effects (Extended Data Fig. 2-2). On a per-dataset basis, MEDiCINe Rigid and MEDiCINe also ranked highest on average among all the methods (Extended Data Fig. 2-3) and did not have extreme failure modes (Extended Data Fig. 2-4).

Prior work has shown that better motion estimation correlates with better spike sorting (Garcia et al., 2024). To verify this, we selected a random set of 40 of our simulated datasets to evaluate spike sorting. For each of these datasets and each motion estimation method, we corrected for the estimated motion in the neural data using Kriging interpolation (Pachitariu et al., 2023) and ran Kilosort4 spike sorting (disabling the built-in motion correction step; Pachitariu et al., 2024). To evaluate sorting quality, we computed a standard metric of spike-sorting accuracy (Garcia et al., 2024; Pachitariu et al., 2024). For any motion estimation method, we define the relative spike-sorting inaccuracy on a dataset A to be as follows:

$$\mathrm{Inaccurac}y_{\mathrm{rel}}(A)\;=\;\mathrm{Inaccuracy}(A)-\underset{B\in estimators}{\min}\;\mathrm{Accuracy}(B),$$

where $\mathrm{Inaccuracy}\;=\;\;\underset{1\leq i\leq N\_\mathrm{neurons}}{\sum }\;(1-\mathrm{Accurac}y_{i})$. MEDiCINe Rigid and MEDiCINe had lower relative spike-sorting inaccuracy than existing methods (Fig. 2*D*; Extended Data Fig. 2-5).

### Neurophysiology datasets

To test MEDiCINe in practice, we used four of our primate Neuropixels sessions with motion artifacts that we found difficult to estimate and correct using existing methods. This data was collected by acute Neuropixels recording of the dorsomedial frontal cortex of awake behaving rhesus macaque monkeys. All experimental procedures conformed to the guidelines of the National Institutes of Health and were approved by the Committee of Animal Care at the Massachusetts Institute of Technology. The recordings exhibited a range of real-world motion and instability conditions. We suspect the primary cause of motion artifacts is movement of the surface of the brain within the recording craniotomy due to changes in intracranial pressure when the animal moves its body. We used monopolar triangular spike localization and applied MEDiCINe to the data. We found that MEDiCINe performed well under these conditions (Fig. 3), qualitatively better than existing methods on these datasets (Extended Data Fig. 3-1).

10.1523/ENEURO.0529-24.2025.f3-1 Figure 3-1 **Non-MEDICINE Results for NHP Datasets.** This shows the results for all non-MEDICINE methods for each of the NHP datasets shown in Figure 3. Download Figure 3-1, TIF file.

10.1523/ENEURO.0529-24.2025.f3-2 Figure 3-2 **Results for Rodent Datasets (A)** Spike raster for one rodent dataset. Note the motion artifacts beginning at 600  s caused my intentional movement of the micromanipulator. **(B)** Plots of the estimated motion (colors) by each method and the motion of the micromanipulator (black), in a time window around the micromanipulator movement. **(C)** Mean absolute error of the estimated motion by each method compared to micromanipulator movement. Download Figure 3-2, TIF file.

In addition to our NHP datasets, we also benchmarked MEDiCINe and existing methods on a rodent Neuropixels dataset with motion imposed by controlled movements of the micromanipulator holding the probe during recording (Steinmetz et al., 2021). On these datasets, we found MEDiCINe to perform at least as well as existing motion estimation methods, when compared with the ground-truth movement of the micromanipulator (Extended Data Fig. 3-2). However, all methods performed similarly on these data. We believe all methods had significant error with respect to the micromanipulator because the micromanipulator motion does reflect the ground-truth motion between the probe and the brain tissue. Specifically, elasticity of the brain tissue and friction between the tissue and the probe cause the micromanipulator movements to be attenuated and smoothed with respect to the brain tissue.

## Discussion

In this work, we introduced a novel method for estimating motion in neurophysiology recordings, called MEDiCINe (Motion Estimation by Distributional Contrastive Inference for Neurophysiology). We found that MEDiCINe outperformed existing methods on an extensive benchmark of simulated datasets with known ground-truth motion. We also found that MEDiCINe performed well on real NHP neurophysiology datasets where existing methods struggle.

There are two key differences between MEDiCINe and existing motion estimation methods (Windolf et al., 2023; Pachitariu et al., 2024). First, MEDiCINe is a probabilistic generative model of the spike data constrained to decompose the data into independent motion and neural activity components. In contrast, existing methods estimate motion by explicitly aligning activity histograms in different time bins throughout the data. Second, MEDiCINe's model of the data is parameterized implicitly, allowing it to leverage the continuity of time, depth, and amplitude, which helps it handle very sparse and noisy data. In contrast, existing methods discretize the data in time, depth, and amplitude, which may cause them to be sensitive to bin sizes and brittle for very sparse or noisy datasets.

We envision several ways to extend and improve the MEDiCINe model:

- LFP features. In this work, we have evaluated MEDiCINe on spike data, but using LFP data may allow it to estimate motion more accurately, particularly for datasets with few neurons. Other works have found LFP features useful for motion estimation (Windolf et al., 2023).
- More spike shape features. In this work, the only spike shape feature we used for motion estimation was amplitude. However, MEDiCINe could readily use other features, such as spike width or waveform shape. In fact, because MEDiCINe uses a sparsity loss based on classification, we expect using more spike features would improve its performance by increasing the sparsity of the motion-corrected time-marginalized spike distribution.
- Fluctuations in firing rate over time. In this work, we used a time-invariant classification network to discriminate dataset spikes from uniformly sampled spikes. However, in practice, neuron firing rates change over time (e.g., due to cell death). Modeling these changes in firing rate could improve the performance of MEDiCINe. One way to do this would be to allow the classification network to depend on time subject to reasonable priors, such as only allowing sparse or slow changes in firing rates.
- Inductive biases on motion. In this work, the motion function *M* was unconstrained aside from a temporal smoothing kernel. This affords the model flexibility, but causes it to sometimes find implausible solutions (Extended Data Fig. 2-4). This could be addressed by incorporating more priors in the motion function, such as a Gaussian process prior on the motion or explicit priors for motion patterns that are likely to occur in neurophysiology data (e.g., discrete jumps and slow monotonic drift).
- Motion in three dimensions. In this work, we only considered motion in the depth direction along the laminar array, not horizontal motion in directions orthogonal to the array. While motion in depth is the most salient and detectable motion axis for laminar arrays, the motion function in MEDiCINe could be directly augmented to model three-dimensional motion, which may offer benefits for users with three-dimensional electrode arrays. This may also allow MEDiCINe to be used for motion estimation in recording modalities other than electrophysiology, such as calcium imaging.

We open-source the MEDiCINe model implementation at https://github.com/jazlab/medicine and provide a website (https://jazlab.github.io/medicine) with documentation, demos, and instructions for installing and using MEDiCINe with just a few lines of code. We also open-source all code and data (https://github.com/jazlab/medicine_paper) necessary for reproducing our results with instructions for how to do so.

## Synthesis

Reviewing Editor: Arvind Kumar, KTH Royal Institute of Technology Decisions are customarily a result of the Reviewing Editor and the peer reviewers coming together and discussing their recommendations until a consensus is reached. When revisions are invited, a fact-based synthesis statement explaining their decision and outlining what is needed to prepare a revision will be listed below. The following reviewer(s) agreed to reveal their identity: Michael Okun.

Synthesis

First of all, apologies for the long delay in processing this manuscript. It was surprisingly difficult to find reviewers who are not connected to one of the authors. And when I managed to find it, one of them went incognito. So I had to make a call on the manuscript based on my own reading and the comments from one of the reviewers.

The manuscript presents an elegant solution to the problem of relative motion of electrodes and the brain tissue. The manuscript clearly presents a methodological advance and is very timely.

Overall the manuscript has a very good structure but I think methods should be made more clear (especially in the main text) and they should be presented in a way a user without much background in contrastive inference can follow the ideas.

On the methodological side, it is important to clearly state the assumptions under which the algorithm works. And specify the kind of motion artifacts that can be captured by the algo.

Comments from the reviewer are appended below. We look forward to reading the revised version of the manuscript.

Reviewer #1

The ms presents a novel method for motion drift detection and correction for Neuropixels (and similar) recordings. The proposed method provides an elegant and efficient solution to the problem - so it is important and timely. I understand that the software is also made publicly available, but since the links to the website were removed from the pdf file by eNeuro (for double blind review), this was not examined. The ms is for the most part easy to read and understand. This software is likely to be of interest to the many labs who perform or analyse silicon probe recordings.

For the final revision I have two key suggestions.

1. Consider performing some testing of the method on rodent recordings, since this is the largest future pool of users and use cases, and what many readers will find most convincing. (I'd leave the final decision on whether to expand their work in this way with the authors).

2. Write the ms to be more accessible. The ms is already well written, however in several places presupposes a rather advanced level of knowledge on the topic. I would suggest to write it in a manner that will be suitable not only for the small number of experts in spikesorting and motion correction algorithms, but also for the wider community of systems neuroscientists who use these tools and would like to understand what they do (and do not want to read a bunch of previous papers). For example, please explain in more detail the major ideas behind your approach, the meaning of the probability P that is computed for each spike, etc. In the Discussion section it would be worthwhile to briefly explain what approaches are taken by Kilosort4 and DREDge, and if there's any intuitive reason to expect MEDiCINe to perform better.

Technical:

• "Historically, spike sorting was a primarily manual process." This was true some 20-25y ago, as more recently it was/is a semi-manual process (some references might be included here).

• Please provide some further details on the example NHP recordings in Fig. 3. Which brain areas were recorded? Was there anything in particular that made these recordings more unstable than others?

• P. 3 "...this matrix at $\Delta D$ " ---> ...this matrix are $\Delta D$

• P. 8 "...in ReLU" ----> is ReLU (or was ReLU)

## Author Response

Your revision must include the manuscript with new text indicated in a bold or highlighted font to aid the re-review of the manuscript. Separately, please provide a clean copy of the manuscript that includes the title page, which will be published if accepted. Please closely review your manuscript at this time for any final corrections in style or substance. Please consult our Revised Submission Checklist for full details on preparing your revision: https://www.eneuro.org/content/revising-finalizing-manuscript#revisions Thank you for these instructions. We followed the submission format guidelines and have submitted Word documents for the revised manuscript and clean copy.

Synthesis of Reviews:

Synthesis Statement for Author (Required):

The manuscript presents an elegant solution to the problem of relative motion of electrodes and the brain tissue. The manuscript clearly presents a methodological advance and is very timely.

Overall the manuscript has a very good structure but I think methods should be made more clear (especially in the main text) and they should be presented in a way a user without much background in contrastive inference can follow the ideas.

On the methodological side, it is important to clearly state the assumptions under which the algorithm works. And specify the kind of motion artifacts that can be captured by the algo.

Thank you for these suggestions. We have updated the methods section to present the method in more accessible language and highlight the assumptions of the method.

Reviewer #1 The ms presents a novel method for motion drift detection and correction for Neuropixels (and similar) recordings. The proposed method provides an elegant and efficient solution to the problem - so it is important and timely. I understand that the software is also made publicly available, but since the links to the website were removed from the pdf file by eNeuro (for double blind review), this was not examined. The ms is for the most part easy to read and understand. This software is likely to be of interest to the many labs who perform or analyse silicon probe recordings.

For the final revision I have two key suggestions.

1. Consider performing some testing of the method on rodent recordings, since this is the largest future pool of users and use cases, and what many readers will find most convincing. (I'd leave the final decision on whether to expand their work in this way with the authors).

Thank you for this suggestion. We have now expanded our work to evaluate and benchmark MEDiCINe and existing methods on open-sourced rodent recordings. We have added Figure S7 in the Supplementary Material to include these results and now also refer to them in the main text.

2. Write the ms to be more accessible. The ms is already well written, however in several places presupposes a rather advanced level of knowledge on the topic. I would suggest to write it in a manner that will be suitable not only for the small number of experts in spikesorting and motion correction algorithms, but also for the wider community of systems neuroscientists who use these tools and would like to understand what they do (and do not want to read a bunch of previous papers). For example, please explain in more detail the major ideas behind your approach, the meaning of the probability P that is computed for each spike, etc. In the Discussion section it would be worthwhile to briefly explain what approaches are taken by Kilosort4 and DREDge, and if there's any intuitive reason to expect MEDiCINe to perform better.

Thank you for these suggestions. We have updated the Methods section to elaborate on the intuition of the model, describe the main idea in clearer words, and explain the meaning of the probability function P. We have also added a paragraph to the Discussion section explaining the key differences between MEDiCINe and existing methods and why it performs better in some conditions.

Technical: • "Historically, spike sorting was a primarily manual process." This was true some 20-25y ago, as more recently it was/is a semi-manual process (some references might be included here).

Thank you, we have clarified this and added references. • Please provide some further details on the example NHP recordings in Fig. 3. Which brain areas were recorded? Was there anything in particular that made these recordings more unstable than others? Thank you, we have added the brain area recorded. We also added our thoughts about what caused the motion artifacts in these recordings. • P. 3 "...this matrix at $\Delta D$ " ---> ...this matrix are $\Delta D$ Fixed, thank you. • P. 8 "...in ReLU" ----> is ReLU (or was ReLU) Fixed, thank you.
