# Supplementary figures and images for: MEDiCINe: Motion Correction for Neural Electrophysiology Recordings
Source: eNeuro. 2025 Mar 4;12(3):ENEURO.0529-24.2025. doi: 10.1523/ENEURO.0529-24.2025 (PMC11896784; doi:10.1523/ENEURO.0529-24.2025)

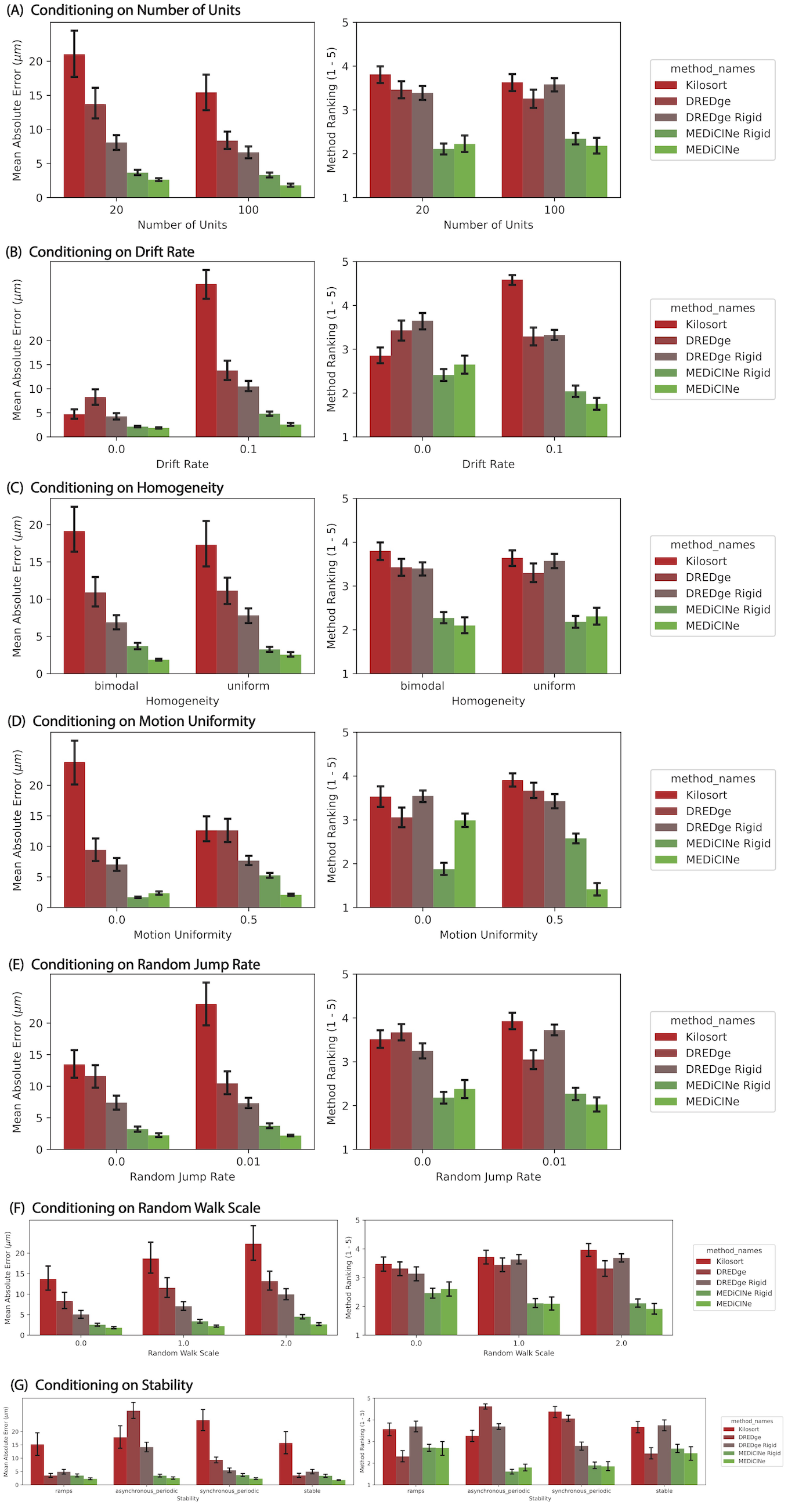

Supplement: Figure 2-1 — Results on Simulated Data Conditioned on Parameters. Motion estimation model results conditioned on each parameter of variation of simulated dataset suite. Errorbars show 95% confidence interval of the mean. Left column shows mean absolute error, and right column shows method ranking. Download Figure 2-1, TIF file. [file eneuro-12-ENEURO.0529-24.2025-s002.tif]

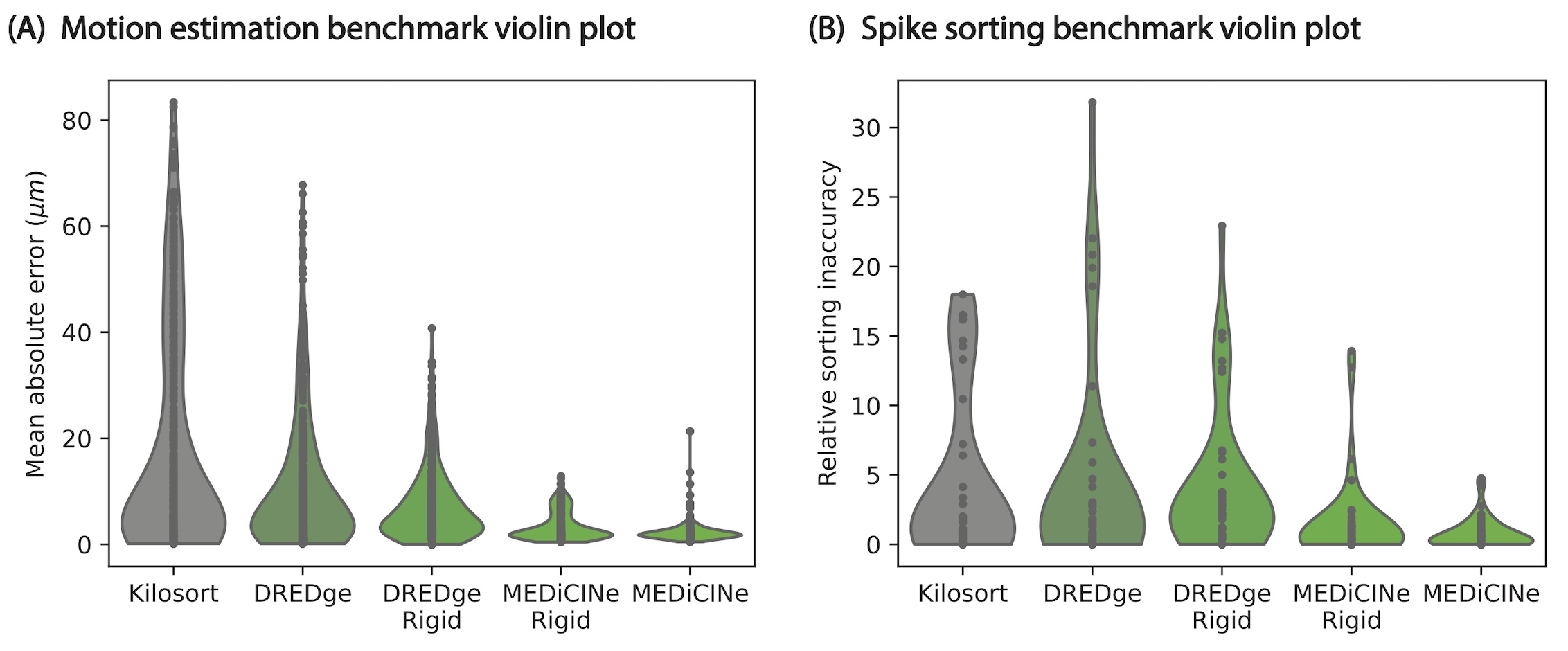

Supplement: Figure 2-2 — Benchmark Violin Plots (A) A violin plot representation of the results in Figure 2-A. (B) A violin plot representation of the results in Figure 2-B. Download Figure 2-2, TIF file. [file eneuro-12-ENEURO.0529-24.2025-s003.tif]

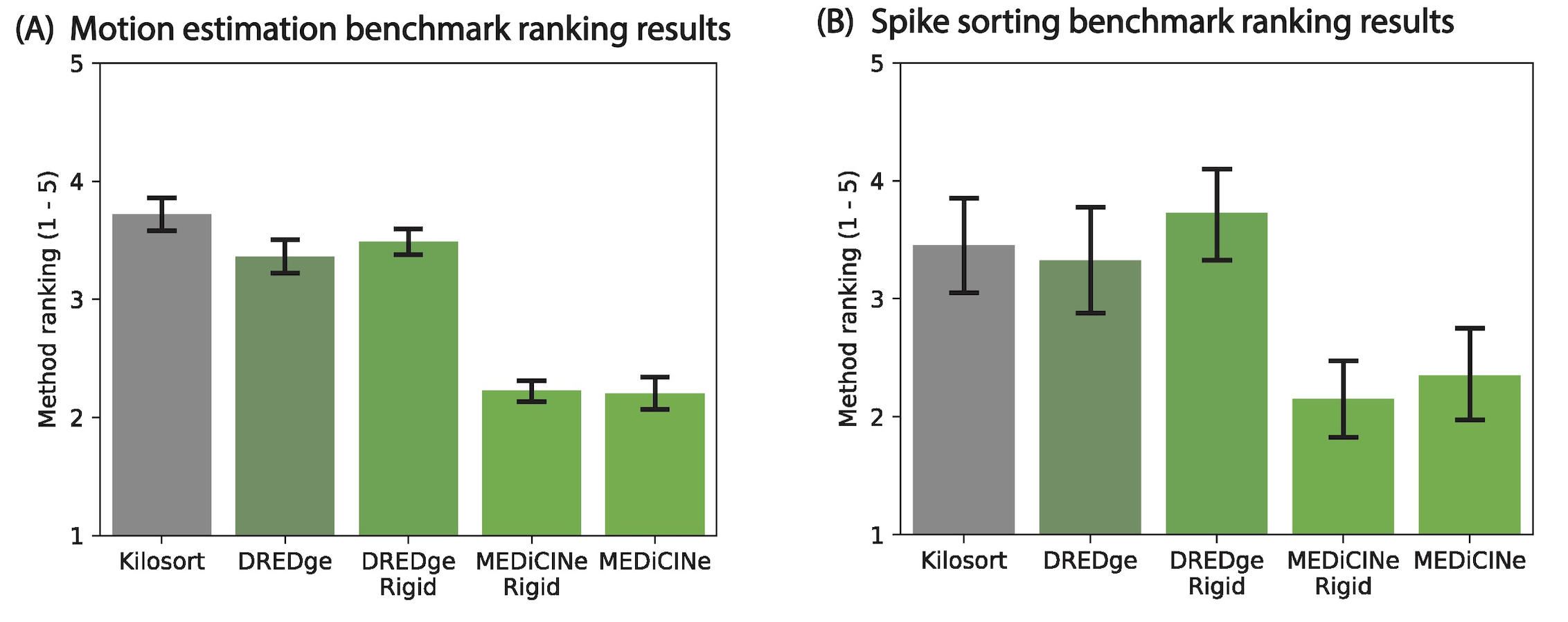

Supplement: Figure 2-3 — Benchmark method Rankings (A) For each simulated dataset, we compute the ranking (1-5) of each of the 5 motion estimation methods on that dataset in terms of mean absolute motion estimation error. This ranking is shown on the y-axis. (B) For each simulated dataset for which we run spike sorting, we compute the ranking (1 - 5) of each of the 5 motion estimation methods on that dataset in terms of relative sorting inaccuracy. This ranking is shown on the y-axis. Download Figure 2-3, TIF file. [file eneuro-12-ENEURO.0529-24.2025-s004.tif]

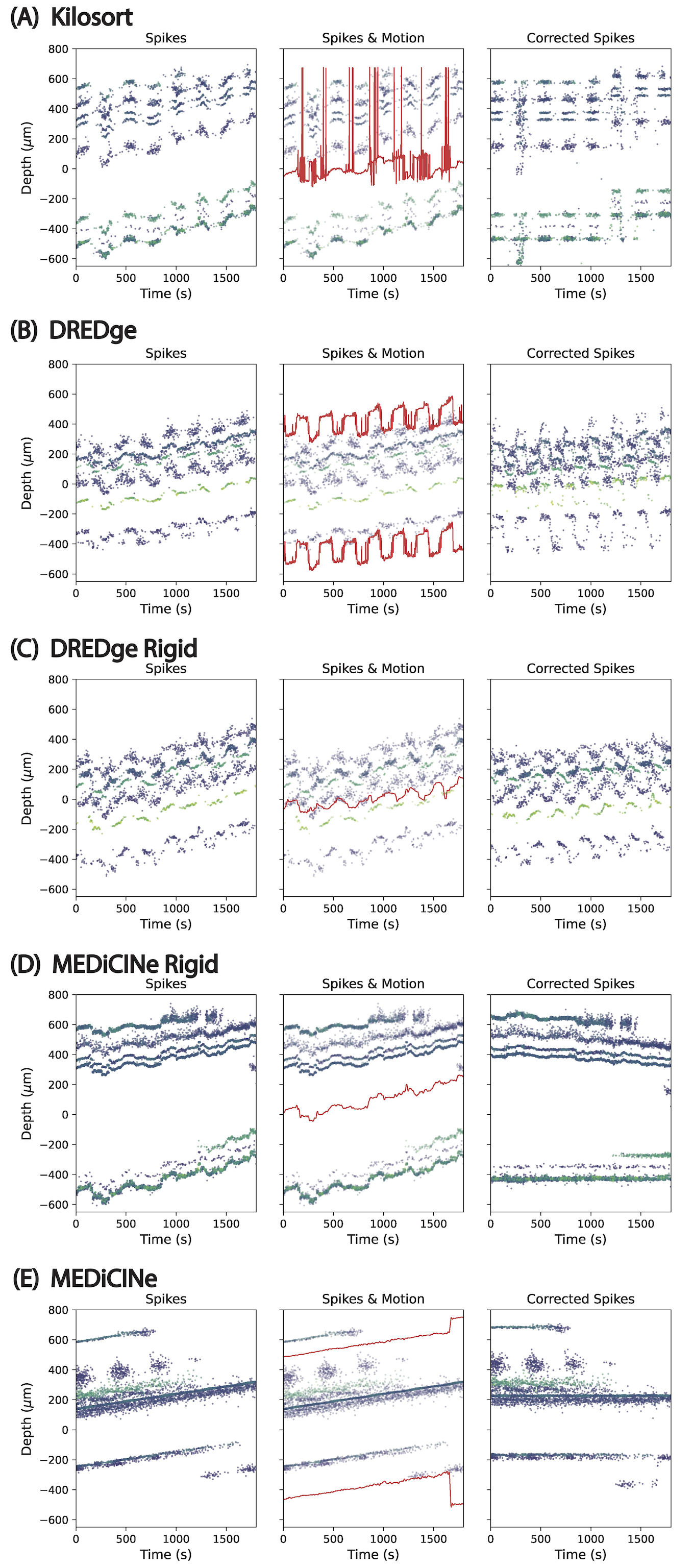

Supplement: Figure 2-4 — Failure Cases (A) Kilosort motion estimation results for the simulated dataset for which the difference between Kilosort and the best method is greatest. This represents the worst failure case for Kilosort in our suite of simulated datasets. (B) - (E) Corresponding failure cases for the other methods. Download Figure 2-4, TIF file. [file eneuro-12-ENEURO.0529-24.2025-s005.tif]

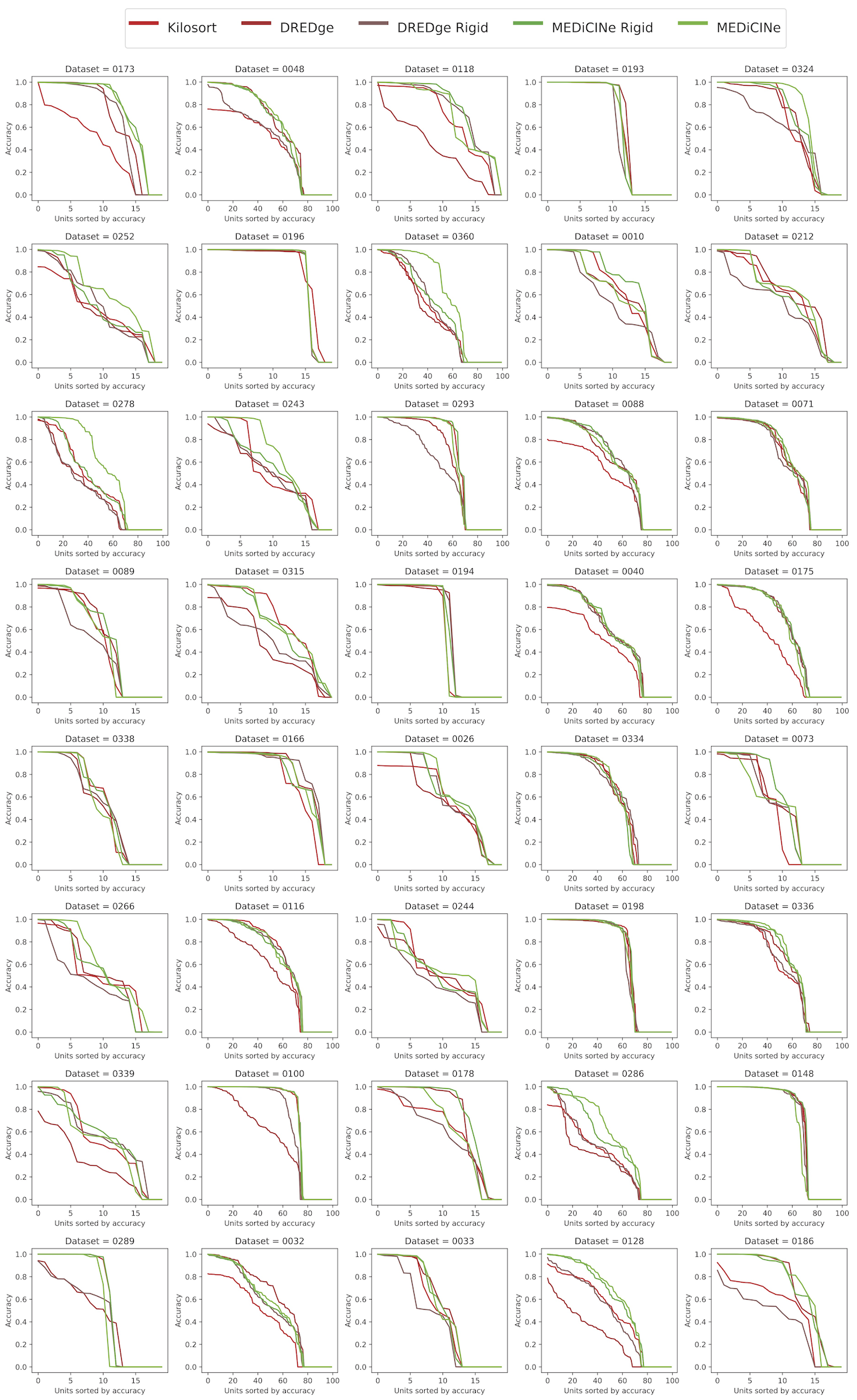

Supplement: Figure 2-5 — Spike Sorting Accuracy. Accuracy as a function of unit (sorted by accuracy) for Kilosort4 sorting results for each motion estimation method on each of the 40 datasets for which we ran spike sorting. Download Figure 2-5, TIF file. [file eneuro-12-ENEURO.0529-24.2025-s006.tif]

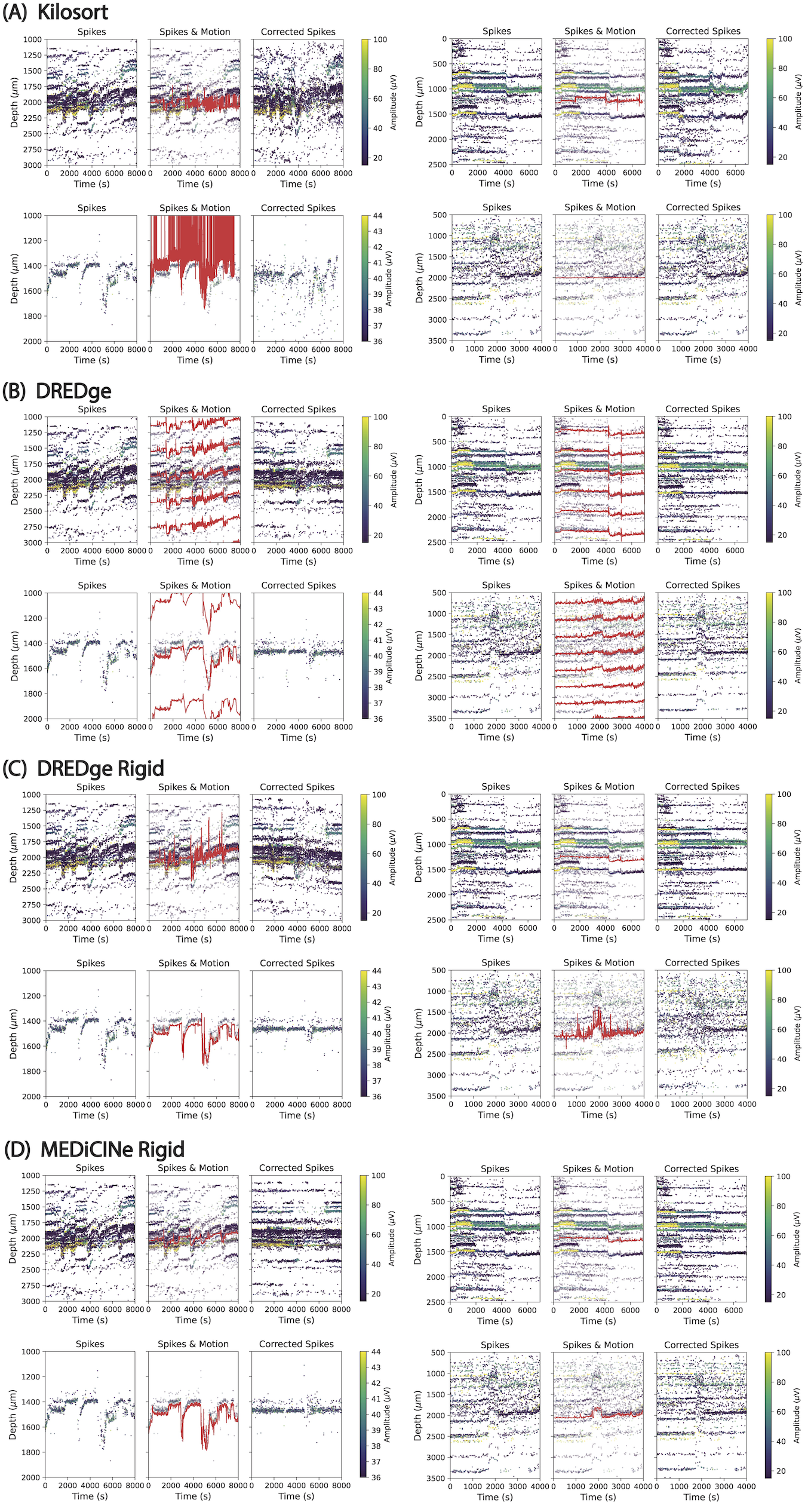

Supplement: Figure 3-1 — Non-MEDICINE Results for NHP Datasets. This shows the results for all non-MEDICINE methods for each of the NHP datasets shown in Figure 3. Download Figure 3-1, TIF file. [file eneuro-12-ENEURO.0529-24.2025-s007.tif]

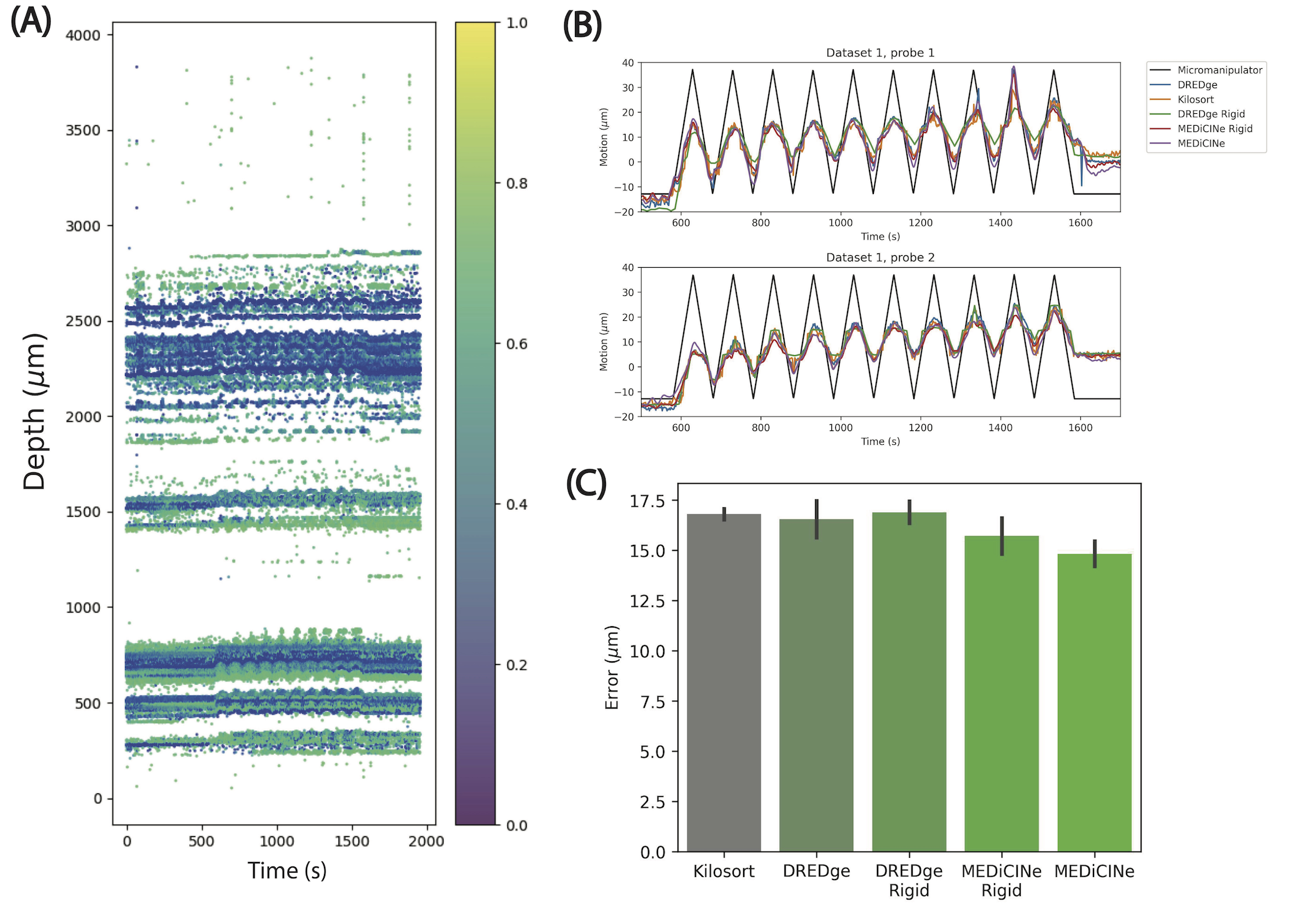

Supplement: Figure 3-2 — Results for Rodent Datasets (A) Spike raster for one rodent dataset. Note the motion artifacts beginning at 600 s caused my intentional movement of the micromanipulator. (B) Plots of the estimated motion (colors) by each method and the motion of the micromanipulator (black), in a time window around the micromanipulator movement. (C) Mean absolute error of the estimated motion by each method compared to micromanipulator movement. Download Figure 3-2, TIF file. [file eneuro-12-ENEURO.0529-24.2025-s008.tif]
